# Heart Break or Headache: A Case Report of Sumatriptan-Induced Takotsubo Syndrome

**DOI:** 10.7759/cureus.45990

**Published:** 2023-09-26

**Authors:** Michael J Valentine, Hunter D Kramer, James Kim, Nicholas Pettinelli, Tom Hu

**Affiliations:** 1 Interventional Cardiology, Kansas City University, Kansas City, USA; 2 Interventional Cardiology, Oklahoma State University, Tulsa, USA

**Keywords:** minoca, migraines, headaches, triptans, sumatriptan, broken heart syndrome, takotsubo syndrome, myocardial infarction with non-obstructive coronary arteries (minoca), myocardial infarction with nonobstructive coronary arteries, stress induced cardiomyopathy

## Abstract

Takotsubo syndrome (TS) describes a transient type of dilated cardiomyopathy that mimics acute coronary syndrome (ACS) on initial presentation. Classic TS presents with marked dilation and ballooning of the left ventricular apex with hyperdynamic basal segments. The most frequent etiology is from emotional and stressful triggers; recently, evidence suggests neurologic and psychiatric involvement. There are increasing reports of TS occurring secondary to migraine abortives. We describe a unique case of TS in a woman after taking sumatriptan to abort her headache.

## Introduction

Takotsubo syndrome (TS) gets its name from the Japanese word for octopus pot, which is a tool used to trap octopus. This pot has a wide base and a slim opening, which resembles the gross appearance of a heart that has been affected by this disorder. Another name for this disorder is stress cardiomyopathy. This is due to the fact that it often occurs in the setting of significant emotional distress, likely due to high concentrations of catecholamines. Indeed, this syndrome relates to the notion of “a broken heart,” as it is often seen in postmenopausal women who have experienced a significant stressor. Of note, this disease commonly presents in the absence of obstructive coronary artery disease (CAD).

Recent evidence concerning the pathophysiology of TS suggests an interplay between neurologic and psychiatric factors [1]. A leading hypothesis of TS postulates that catecholamine exerts a major role [1,2]. There have been few cases that have reported TS secondary to triptan class administration, with sumatriptan as the most frequent drug of class to be mentioned [3-6].

## Case presentation

A 57-year-old female with a history of migraines without aura, depression, and anxiety presented with left-sided chest pain, which radiated to her back. Social history was significant for a six-pack-year smoking history. She had a blood pressure of 150/98, heart rate of 78, respiratory rate of 22, temperature of 36.8 C, and oxygen saturation of 97% on room air. A cardiovascular exam revealed normal S1 and S2, and a regular rate and rhythm, without murmurs, rubs, or gallops.

**Figure 1:**
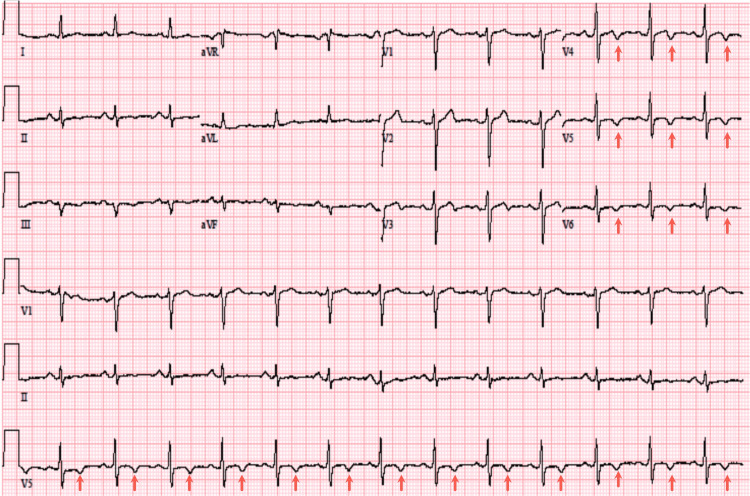
Twelve lead electrocardiogram demonstrating lateral T-wave inversion in leads V4-V6

**Figure 2:**
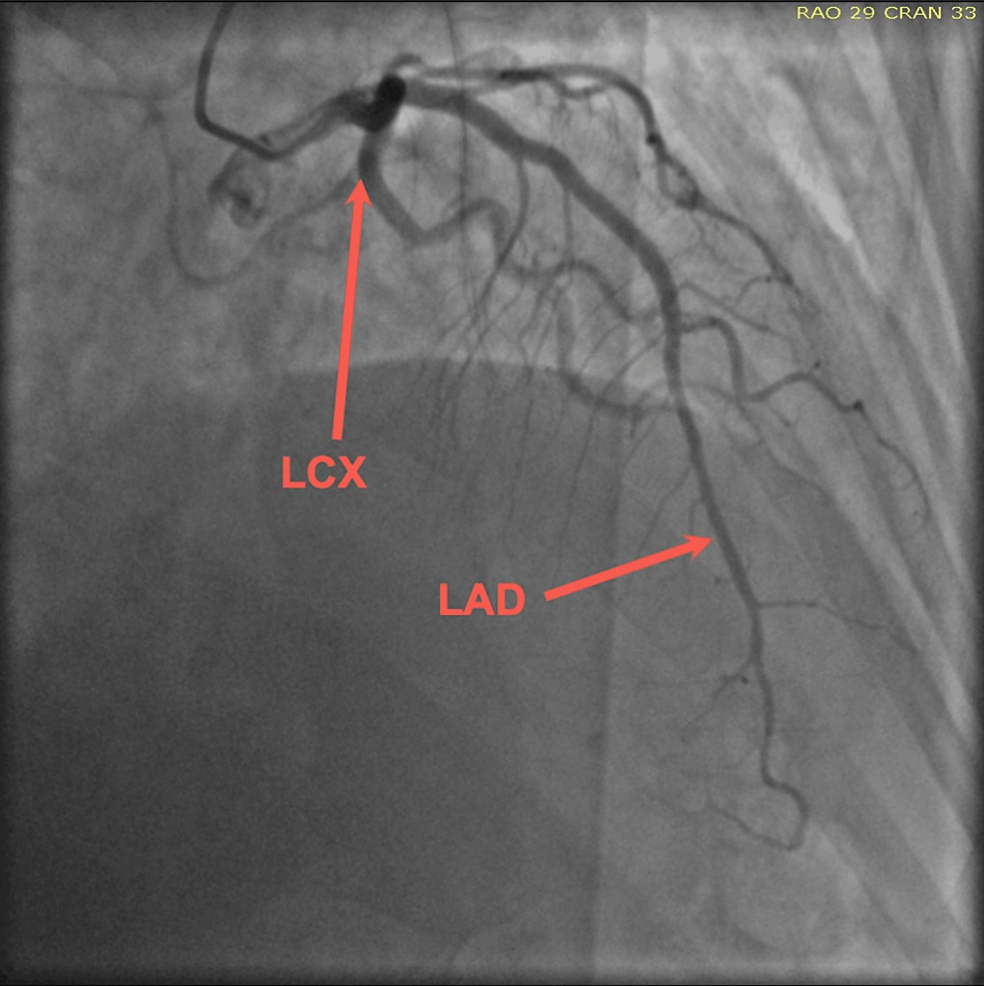
Coronary angiography (PA/CRA) demonstrating no significant coronary disease of the LAD and LCX PA, postero-anterior; CRA, cranial; LAD, left anterior descending artery; LCX, left circumferential artery

**Figure 3:**
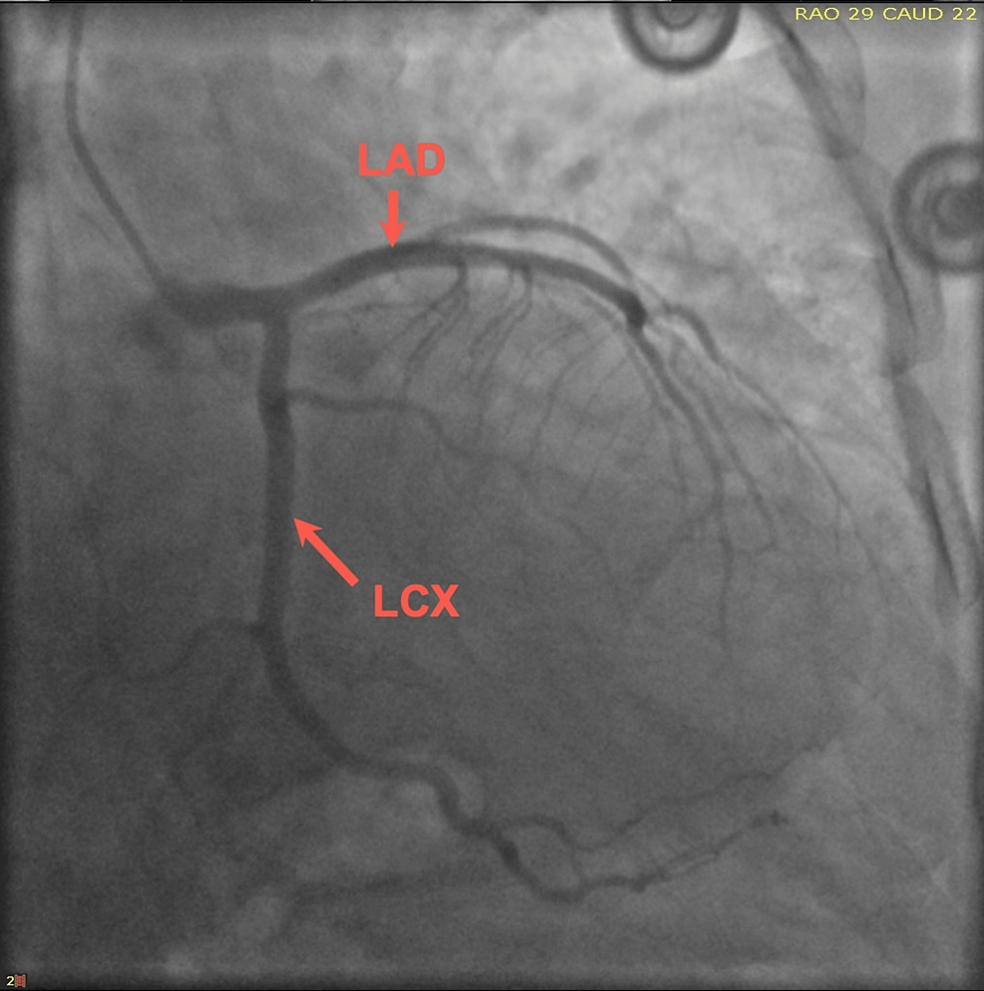
Coronary angiography (RAO/CAU) demonstrating no significant coronary disease of the LAD and LCX RAO, right anterior oblique; CAU, caudal; LAD, left anterior descending artery; LCX, left circumferential artery

**Figure 4:**
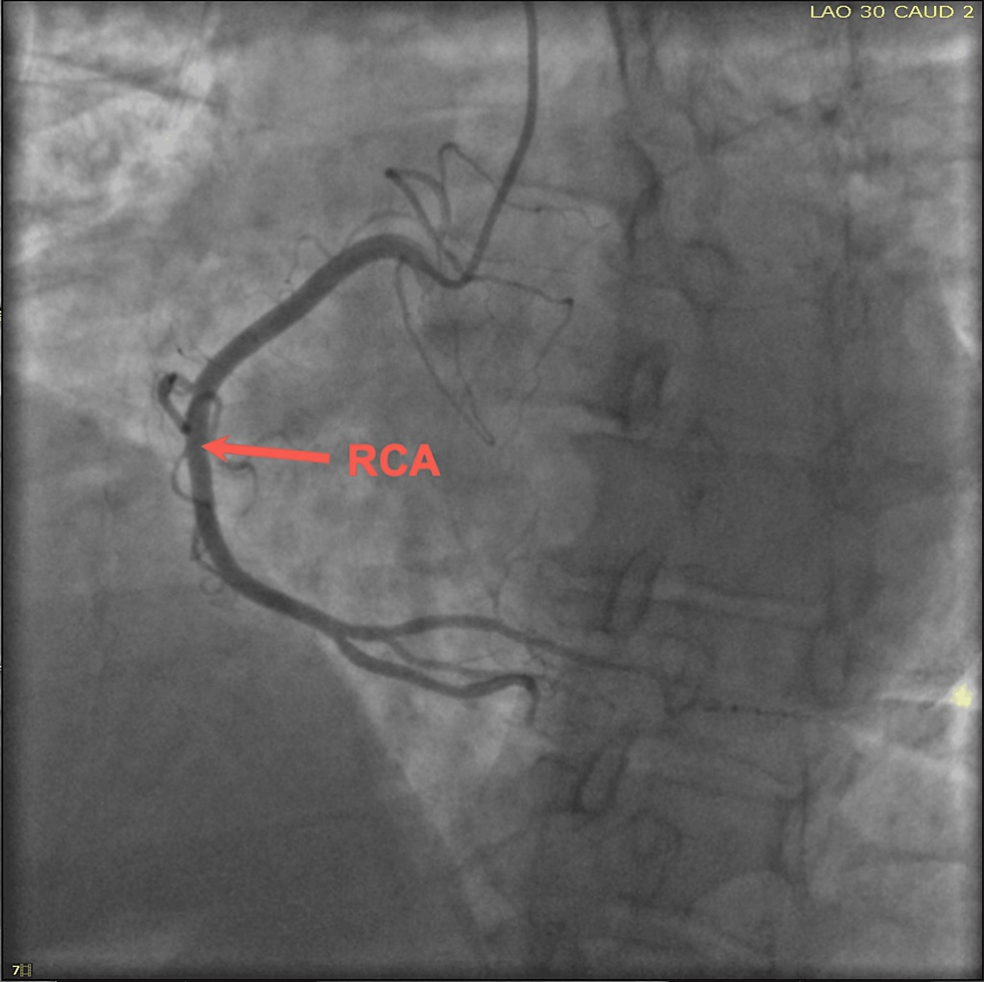
Cardiac angiography (LAO/CAUD) demonstrating no significant coronary disease of the RCA LAO, left anterior oblique; CAUD, caudal; RCA, right coronary artery

**Figure 5:**
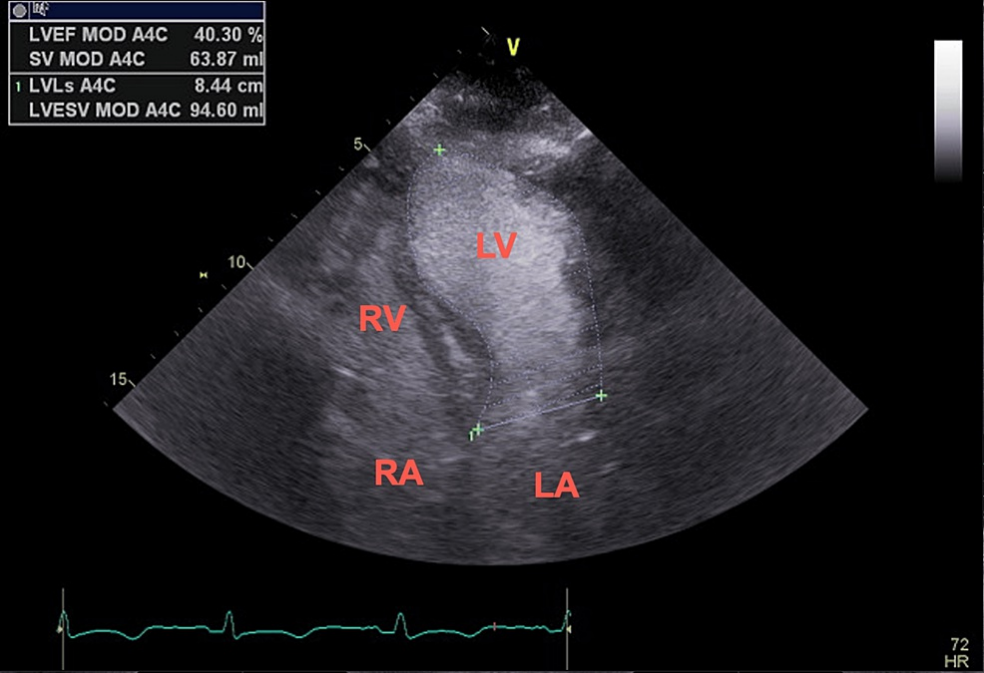
Transthoracic echocardiogram (apical four-chamber view) demonstrating depressed ejection fraction at 40% by Simpson’s method with apical hypokinesis at end-systole RV, right ventricle; LV, left ventricle; RA, right atrium; LA, left atrium

**Figure 6:**
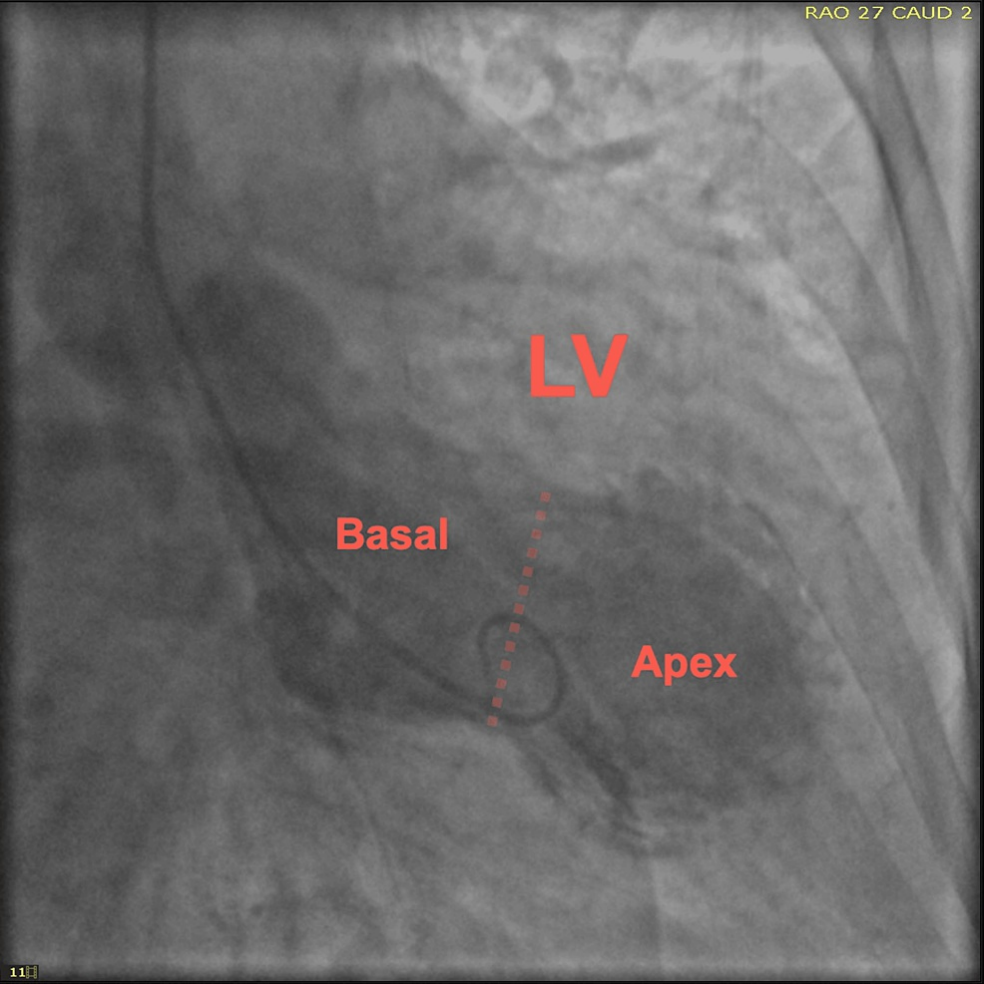
Left ventriculogram demonstrating apical hypokinesis with basal hyperkinesis LV, left ventricle; RAO, right anterior oblique; CAUD, caudal

Upon her arrival, the patient’s initial ECG demonstrated anterior lateral T-wave inversion (Figure 1), along with an elevated troponin of 0.82 ng/mL. Notably, the patient denied recent stressful events. Consequently, a thorough investigation revealed that she had taken sumatriptan earlier that morning to treat a migraine. The patient underwent a coronary angiogram due to suspicion of acute coronary syndrome (ACS) and demonstrated no significant coronary disease in the left anterior descending artery (LAD), circumferential artery (Cx), and right coronary artery (RCA) (Figures 2-4). Transthoracic echocardiogram (Figure 5) and left ventriculogram (Figure 6) revealed a 40% reduced ejection fraction and classic presentation of mid to apical akinesis and basal hyperkinesis.

The patient tolerated the procedure well and was initiated on a treatment regimen involving metoprolol succinate and lisinopril. She was instructed to discontinue sumatriptan and seek an alternative migraine abortive regimen. The patient was discharged with a plan for a repeat outpatient echocardiogram.

## Discussion

TS is a reversible cause of nonischemic cardiomyopathy. TS symptoms often mimic ACS with chest pain, shortness of breath, diaphoresis, and lightheadedness. ACS can be distinguished from TS via cardiac catheterization, which will show the findings of basal hyperkinesis and apical akinesis. Due to the relatively new discovery of this rare disease in the 1990s and the abundance of misconceptions, prevalence is difficult to estimate. Napp and Bauersachs suggest that this syndrome is underdiagnosed [1]. Interestingly, they also concluded that TS incidence is higher in patients with a history of neurological disease. 

The mechanism of TS remains largely unknown. The most promising hypothesis of the TS mechanism theorizes catecholamine overload as the main pathophysiologic mechanism [1,2]. Yet, a valid limitation to the catecholamine theory suggests that if catecholamines were truly the underlying driving factor in the progression of TS, then we would likely see a surge in TS incidence globally, given the copious number of patients receiving catecholamines worldwide. Therefore, catecholamine cannot have a causative role but may contribute a significant triggering role. Regardless, sympathetics seem to bear importance in TS pathology but remain inconclusive. Uniquely, recent evidence suggests an underlying brain-heart axis requirement that is implicated in TS development [1].

Treatment of TS is based on the treatment principles of systolic heart failure, which involves controlling pulmonary congestion with diuretics, arterial vasodilators, and beta blockers. Notably, clinicians should pay attention to possible dynamic outflow obstruction. Catecholamines and inotropes should be avoided in that setting due to increased Venturi’s effect and worsening hemodynamics due to dynamic outflow obstruction. Careful initiation of beta blockers and fluid hydration can relieve the outflow obstruction. 

There are very few cases that report TS secondary to medications. Sumatriptan is the most common migraine abortive that has been mentioned to be associated with TS induction [3,4]. One case has reported zolmitriptan induction, and another case has reported rizatriptan [5,6]. This seems to suggest the implication of triptan class in the induction of TS, albeit with very limited data. Therefore, some clinicians may consider exploring the replacement of triptan drugs with a migraine abortive alternative such as calcitonin gene-related peptide or its receptor (CGRP(r)mAbs) monoclonal antibodies or gepants [7]. 

Current theory regarding migraines points to vasodilation. The mechanism in which triptans abort acute migraines is by elicitation of vasoconstriction via binding to serotonin 5-HT1 receptors [8]. TS induction from sumatriptan is theorized to occur from coronary vasospasm in the circumstance of acute migraines due to an already elevated sympathetic state [3]. Our case seems to agree with this suggestion. Conversely, due to the rarity of TS’s induction and unknown pathophysiology, more research is needed to definitively conclude the association between the triptan drug class and TS.

## Conclusions

TS is becoming increasingly recognized as an important etiology of dilated cardiomyopathy. However, the exact mechanism of TS remains elusive. The most accepted hypothesis is the overload of catecholamine and its associated myocardial stunning. Sumatriptan, among the triptan class, is the most frequent drug of the class mentioned to be associated with TS. There is rising evidence that sumatriptan is not the only one of its class to trigger induction, but it remains unresolved. This case highlights that a thorough medication review is critical to uncover novel and vital connections that germinate future investigations of TS.

## References

[1] Napp LC, Bauersachs J (2020). Takotsubo syndrome: between evidence, myths, and misunderstandings. Herz.

[2] Nef HM, Möllmann H, Weber M, Deetjen A, Brandt R, Hamm CW, Elsässer A (2007). Release pattern of cardiac biomarkers in left ventricular apical ballooning. Int J Cardiol.

[3] Rathore SA, DeLeon D (2014). A variant form of takotsubo syndrome secondary to Sumatriptan: a case report. Case Rep Intern Med.

[4] Mohan J, Parekh A, DeYoung M (2019). Sumatriptan induced takotsubo cardiomyopathy; the headache of the heart: a case report. Front Cardiovasc Med.

[5] Garg J, Aronow WS, Devabhaktuni S, Ahmad H (2015). Takotsubo syndrome (or apical ballooning syndrome) secondary to zolmitriptan. Am J Ther.

[6] Somers-Edgar TJ, Shah J, Kueh A, Kasargod Prabhakar C (2023). Triptan-induced takotsubo syndrome: a case report. Eur Heart J Case Rep.

[7] Lampl C, MaassenVanDenBrink A, Deligianni CI (2023). The comparative effectiveness of migraine preventive drugs: a systematic review and network meta-analysis. J Headache Pain.

[8] MacIntyre PD, Bhargava B, Hogg KJ, Gemmill JD, Hillis WS (1993). Effect of subcutaneous sumatriptan, a selective 5HT1 agonist, on the systemic, pulmonary, and coronary circulation. Circulation.

